# Mosaic Origins of a Complex Chimeric Mitochondrial Gene in *Silene vulgaris*

**DOI:** 10.1371/journal.pone.0030401

**Published:** 2012-02-27

**Authors:** Helena Storchova, Karel Müller, Steffen Lau, Matthew S. Olson

**Affiliations:** 1 Institute of Experimental Botany, Academy of Sciences of the Czech Republic, Prague, Czech Republic; 2 Institute of Arctic Biology and Department of Biology and Wildlife, University of Alaska Fairbanks, Fairbanks, Alaska, United States of America; 3 Department of Cell Biology, Max Planck Institute for Developmental Biology, Tübingen, Germany; 4 Department of Biological Sciences, Texas Tech University, Lubbock, Texas, United States of America; Boston University, United States of America

## Abstract

Chimeric genes are significant sources of evolutionary innovation that are normally created when portions of two or more protein coding regions fuse to form a new open reading frame. In plant mitochondria astonishingly high numbers of different novel chimeric genes have been reported, where they are generated through processes of rearrangement and recombination. Nonetheless, because most studies do not find or report nucleotide variation within the same chimeric gene, evolution after the origination of these chimeric genes remains unstudied. Here we identify two alleles of a complex chimera in *Silene vulgaris* that are divergent in nucleotide sequence, genomic position relative to other mitochondrial genes, and expression patterns. Structural patterns suggest a history partially influenced by gene conversion between the chimeric gene and functional copies of subunit 1 of the mitochondrial ATP synthase gene (*atp1*). We identified small repeat structures within the chimeras that are likely recombination sites allowing generation of the chimera. These results establish the potential for chimeric gene divergence in different plant mitochondrial lineages within the same species. This result contrasts with the absence of diversity within mitochondrial chimeras found in crop species.

## Introduction

Chimeric genes are formed through processes of recombination and duplication that result in the fusion of segments derived from DNA fragments in different genomic positions. Because processes generating chimeric genes can result in novel protein structures, they are significant sources of evolutionary innovation in humans, insects, and plants [1], [2], [3], [4]. Formation of adaptive chimeric genes in plant mitochondria has been well known for over 2 decades [5] and is related to the unique tempo and mode of evolution of the plant mitochondrial genome. Unlike animal mitochondria, in most flowering plant genera mitochondrial coding regions evolve very slowly relative to those in chloroplast and nuclear genomes [6], [7], [8], [9], whereas rearrangements and intragenomic recombination can be quite dynamic, even within species [10], [11], [12], [13]. Patterns of rearrangement and duplication within plant mitochondria are complex, but can be conceptually categorized into common and rare events [10], [11], [14]. Common rearrangements are associated with large repeats in the mitochondrial genome (>4 kb in Arabidopsis [15]). These generate mini-molecules, which are in constant flux and can be assembled into mitochondrial master molecules according to the Multipartite model [8], [16],[17]. Rearrangements associated with shorter repeats (<500 bp) are much less common, perhaps because nuclear surveillance loci such as *MSH1* inhibit development of the new conformation of DNA [18], [19], [20]. These rare recombination processes may occasionally produce novel chimeric genes that are expressed and have phenotypic effects in mature plants. The rate at which rare rearrangements arise is currently unknown, and it is unlikely that all chimeric genes are expressed; therefore the rate at which expressed chimeric recombinants arise is likely even lower.

Because the creation of each chimeric gene involves unique situations, each gene is assumed to have originated only once. The fate of these genes, however, is mysterious because few studies report allelic variation within chimeric genes (but see [21]). Chimeric mitochondrial genes composed of the same genic segments are not shared across plant species, suggesting that they do not persist long in the mitochondrial genome. Also, multiple different mitochondrial chimeric genes have been identified in several crop species (e.g. maize [1]). These patterns support a process whereby some mitochondrial chimeric genes originate and go extinct at a rate faster than speciation occurs.

Species with gynodioecious breeding systems that carry mitochondrial chimeric genes controlling male-sterility exhibit higher diversity at mitochondrial housekeeping genes (chimeric genes were not studied) than congeners with hermaphroditic breeding systems [22]. This pattern is consistent with longer-term maintenance of the same mitochondrial types within gynodioecious species, perhaps due to balancing selection maintaining cytoplasmic male sterility (CMS). Insight into the processes generating chimeric genes has been gained through studies of CMS genes, which act to block the production of viable pollen and render female an otherwise hermaphroditic plant. CMS genes are usually composed of segments of mitochondrial housekeeping genes such as *ATP synthase* or *cytochrome oxidase* subunits that are spliced together with a region of unknown origin (reviewed in [1]). Transcriptional start positions and regulatory regions are recruited from the housekeeping genes [23], [24], thus the regulatory region does not have to evolve *de novo*. Chimeric CMS genes from different species, and even within the same species, do not share structural homology, reflecting their independent generation [1]. The presence of the region of unknown origin underscores the unique origin of each CMS gene because these unknown regions cannot be found in mitochondria that lack the particular CMS gene. Therefore, it is assumed that the proper circumstances for the origination of a new CMS must be fleeting.

**Figure 1:**
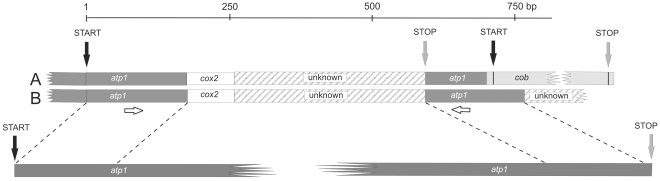
Structure and genomic context of the two variants of *bobt*, *bobt_KR* (A) and *bobt_MV* (B). *bobt_KR* and *bobt_MV* differ in 43 nucleotide sites, but share the same general *atp1-cox2*-unknown ORF structure between the start and stop sites. They share a segment of *atp1* at the 3′ end downstream of the stop codon, a segment of *cox2*, and a segment of unknown origin. The fourth segment, located just downstream of stop codon corresponds to atp1. Its size differs between both copies, because *bobt_KR* is adjacent to *cob*, whereas *bobt_MV* is not. Horizontal arrows designate the locations of the primers (atp1 lo, atp1 up) originally used to discover *bobt* via PCR co-amplification with the functional copy of *atp1*. A full-length *atp1* gene is shown below the *bobt* variants, the homologous regions are indicated by dotted lines. A scale in bp is given above.

The genus *Silene* is the subject of numerous studies of mitochondrial genomics and the population genetics and evolution of mitochondrial genes in natural populations [25], [26], [27], [28], [29]. Here we document a chimeric mitochondrial open reading frame that was first identified in *Silene vulgaris* when it was PCR-amplified along with *ATP synthase 1* (*atp1*) when using primers designed to amplify *atp1* (Fig. 1). The initial fragment, the transcribed RNA, and the gene have been named “*bobt*.” *Bobt* is not present in all individuals. An analysis of the within and among population patterns of *S. vulgaris* plants that carried the *bobt* PCR fragment in a small region of western Virginia can be found in [28] and [30], wherein *bobt* co-segregates with haplotype *b*.

Here we analyze differences between two allelic versions of the mitochondrial chimeric gene *bobt* with highly divergent nucleotide sequences and expression variation, a unique phenomenon that has not yet been reported. We also show that one version of this chimeric gene is co-transcribed with *cytochrome b* (*cob*), whereas the other version is not adjacent to another coding region. Finally, qRT-PCR experiments indicated that these chimeric genes exhibit significantly higher transcription levels in female plants than in hermaphrodites, which supports the candidacy of at least one variant as a CMS gene. These discoveries indicate that mitochondrial chimeric genes have residence times in natural populations that are sufficiently long to allow nucleotide and expression divergence among alleles.

## Methods

### Plant material and PCR screening

A small sample of *Silene vulgaris* plants grown from seed were screened for the presence of *bobt* using a PCR assay described below. These seeds were collected from natural populations in Virginia, Austria, Germany, and the Czech Republic; collection sites were the same as in [28] and [31]. Additional seeds were sent to us by Dr. David McCauley from 2 populations in New York, two in Vermont, and one population in Broadway, Virginia; locations of these sites can be found in [32]. Finally, seeds were sent to us by Dr. Natalia I. Tiupitzina from a site near Krasnoyarsk, Russia. All collections were made along roadsides in public right of ways or public areas that were not subject to permitting. The field studies also did not involve endangered or protected species. Plants were propagated from seed in the greenhouse in pots filled with perlite, vermiculite and coconut coir (1∶1∶1) and fertilized 2–3 times per week depending on the season. To ensure controlled growth conditions during expression studies for plants from Mountain View Virginia (MV) and Krasnoyarsk, Russia (KR), we transferred the plants to a Percival growth chamber and cultivated them in a 19 h light/5 h darkness photoperiodic regime (23°C, 80% humidity, light intensity 80 µM/m^2^/sec).

Genomic DNA was isolated from fresh leaf material using either Qiagen Plant Mini kits or according to [33]. The presence or absence of *bobt* in *Silene vulgaris* individuals from natural populations can be detected as a 2-band pattern when PCR-amplified with *atp1* primers 1 and 2 (Table S1); one band is *atp1* and the second band is *bobt*. All individuals were screened for the presence of *bobt* using this PCR assay.

### Southern hybridization

For Southern analysis, 1 µg of genomic DNA was digested with restriction enzyme *EcoRI*, electrophoresed overnight on a 0.7% agarose gel, and transferred to a positively charged membrane (Hybond N+, Amersham) by capillary blotting. A 1.5 kb fragment of the *cox1* gene, a 1.4 kb fragment of the *atp1* gene and a 220 bp fragment of the *bobt* gene were amplified and labeled with digoxigenin (DIG) using PCR labeling kit (Roche) according to the manufacturer. The PCR *bobt* probe included a portion of the ORF of unknown origin, which was not similar to any sequence available in GenBank. The primers used to generate the probes are provided in Table S1. The membranes were hybridized with DIG-labeled probes and visualized as described by [31].

### Gene expression estimation using qRT PCR

Total RNA extracted by means of RNeasy Plant Mini Kit (Qiagen) was treated with DNase I (Ambion, Europe). One µg of DNAse I treated total RNA and 3.22 µg of random hexamers (Roche Applied Science, Germany) were heated 10 minutes at 65°C, chilled on ice and mixed with RT buffer, 0.5 µl of Protector RNase Inhibitor (Roche Applied Science, Germany), 1.2 µl of 10 mM dNTPs and 40 units of Transcriptor Reverse Transcriptase (Roche Applied Science, Germany), final volume 25 µl. The first strand cDNA was synthesized at 50°C for 30 min. RNA samples were reverse transcribed in two independent RT reactions and each cDNA was measured twice. All repeated measurements were consistent.

First strand cDNA was diluted 10× and qPCR was performed using the Light Cycler 480 SYBR Green I Master (Roche Applied Science, Germany) in a final volume of 10 µl with 300 nM of each of the HPLC purified primers (Table S1) supplied by Metabion. The LightCycler LC 480 (Roche Applied Science, Germany) was programmed as follows: 10 min of initial denaturation at 95°C, then 40 cycles for 10 s at 95°C, 10 s at 58°C, and 15 s at 72°C. PCR efficiencies were estimated from calibration curves generated from serial dilution of cDNAs. The relative ratio of the target gene was calculated as follows:
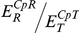
Where E_T_/E_R_ represents the efficiency of target/reference amplification and CpT/CpR represents cycle number at target/reference detection threshold (crossing point). A negative control consisting of PCR mixture with RNA instead of cDNA was performed for each RNA sample to confirm the efficiency of the DNAseI treatment. Transcript levels were normalized with mitochondiral (mt) 18S rRNA of *S. vulgaris*. The invariant levels of this reference transcript were confirmed by direct quantification of cDNA, as described by [34]. A t-test implemented in Excel was used to estimate significance of transcript level differences between hermaphrodites and females.

Finally, quantitative PCR was adopted to compare the abundance of genomic regions under the same conditions as qRT PCR with total DNA used as a template instead of cDNA.

### Inverse PCR

Seven µl of genomic DNA solution (70 ng/µl) was digested with EcoRI or HindIII in 50 µl final volume. The digestion mixture was heat inactivated, 10× diluted and ligated with 8 units of T4 ligase (MBI Fermentas) in 100 µl final volume at 5°C overnight. These conditions supported self-ligation of EcoRI fragments [35]. A self-ligated fragment was digested by HindIII or BglII to provide a linear template for the subsequent PCR amplification. PCR was performed in Biometra T Gradient thermocycler with 5 µl of ligation mixture, 0.16 mM each of primers, 2.5 mM MgCl_2_, 320 mM dNTP and 1 unit *Taq* DNA polymerase (Promega) per reaction. PCR conditions were: 2 min at 94°C; 36 cycles for 1 min at 93°C, 1 min at 58°C and 2 or 3 min at 72°C, with a final extension of 5 min at 72°C. The resulting fragments were cloned in pGEM-T Easy vector (Promega) and sequenced.

### Sequencing of *bobt* and *atp1*

The sequencing strategy is depicted in Figure S7. The central portion of *bobt* was amplified and sequenced using primers **1** and **2** (Table S1). The 5′ and 3′ portions of *bobt_MV* were amplified and sequenced from the product of inverse PCR of HindIII or EcoRI fragment with the primers **8** and **9**. The sequence was confirmed by resequencing using primers targeted to regions specific for *bobt* (**18/22** and **21/2**). The 5′ and 3′ portions of *bobt_KR* were sequenced in the same manner, but using different primers: **6** and **7**. Sequence of the *cob* gene adjacent to *bobt_KR* was obtained on the basis of PCR fragment generated by primers **3** and **5** using additional internal primers. The sequence of an entire *bobt_KR*-*cob* region was confirmed by using specific primers **10/2, 3/26** and **4/5** (Table S1)

The *atp1* variants (except their short 3′ extremities) were amplified using specific primers (Table S1). The *atp1.1_KR* variant was amplified using the primers **16** and **17**, *atp1.2_KR* by the primers **10** and **11** *atp1.1_MV* by the primers **18** and **17**, *atp1.2_MV* by the primers **18** and **20**. Internal primers **12** and **13** were applied to fill the gaps. To sequence 3′ ends of *atp1* variants the primers **14** and **15** were employed. To provide resolution within the *Silene vulgaris* clade, *atp1.2* was sequenced in two individuals that originated form a population near Kovary, Czech Republic. *Atp1.1* was not found in these individuals from Kovary.

The sequences from this study were deposited in GenBank under accession numbers HQ437988-437995 and JF343540-343541.

### Phylogenetic analyses

Phylogenetic analyses were conducted in GARLI v0.96 [36], which simultaneously searches for the topology and tree lengths that maximize the likelihood of the data. The nucleotide substitution model was determined using Akaike Information Criteria using a correction for small sample size (AICc) in JMODELTEST v 0.1.1 [37], [38]. Optimal mutation models used for maximum likelihood analyses differed among data sets. For both the complete *atp1* dataset and the *bobt-atp1* homologous region data set, TPMuf+G [39] had the lowest AIC, so this model was used for all analyses. Six replicate runs were conducted for each dataset with random starting trees. Each replicate was run for 20,000 iterations. To determine clade support, 1000 ML bootstrap replicates were produced in GARLI, each was run for a length of 3,000 iterations. To check the results maximum likelihood analyses also were conducted in PAUP* 4.0b [40]. Results from GARLI and Paup* analyses were identical.

Partition homogeneity tests were implemented in PAUP* to compare the phylogenetic signal from two regions of *bobt* with homology to *atp1*. The null hypothesis for a single origin of the *bobt_MV* and *bobt_KR* genes was tested using Kishino-Hasegawa [41] and Shimodaira-Hasegawa [42] tests implemented in PAUP* 4.0b [40].

## Results

### Complete sequence of *bobt* in two genomes

Within a small haphazard sample of plants originating from the eastern US, Europe, and eastern Russia screened using a PCR based assay, we found the chimeric *bobt* gene only in plants from the region around Mountain Lake, Virginia and in plants from Krasnoyarsk, Russia. Further investigation of the Virginian and Russian copies of *bobt* using PCR-amplification with *atp1*-specific primers, inverse PCR, and direct sequencing revealed two divergent versions (alleles) of the *bobt* gene from the two regions: Mountain View, Virginia (*bobt_MV*) and Krasnoyarsk, Russia (*bobt_KR*). Although they shared the same overall structure and are composed of homologous genic segments, the two *bobt* alleles differed considerably in nucleotide sequence and genomic context. The structure of the *bobt* coding region was composed of three segments: a 5′ 192 bp segment with homology to *atp1*, an 81 bp segment with homology to *cox2*, and a region of 443 bp with unknown homology (Fig. 1). An 8 bp nucleotide region at the junction of the *atp1*- and *cox2*-derived portions of *bobt* shared homology with both genes and is a likely region for intragenomic recombination that generated the chimera. The nucleotide sequences between the start and stop codons of *bobt_MV* and *bobt_KR* differed in 37 positions (8.4% of the sites; Fig. S1); 20.5 of these were at synonymous sites (Ks = 0.13) and 16.5 were at nonsynonymous sites (Ka = 0.03; Fig. S1). The regulatory/promoter region upstream from the start codon was composed primarily of mono- and di-nucleotide repeats, with *bobt_MV* and *bobt_KR* sharing a region that included a 16 bp motif similar to the conserved region in angiosperm *atp1* 5′UTR (positions −181 to −166 Fig. S1, [43]). The primary difference between the regions 5′ to *bobt_MV* and *bobt_KR* was the presence of an 8 bp sequence (ATTTTAAT) in *bobt_MV*, which was shared with the MV copy of *atp1.2* (positions −9 to −24 in the alignment). Following the stop codon was a 3′ segment with homology to *atp1*; in the KR version this region was 85 bp, whereas in the MV version this region was 163 bp (Fig. 1). The KR version of *bobt* was located directly 5′ to the *cob* gene, whereas *cob* was not in this position for the MV version (Fig. 1).

### Inheritance of *bobt*

We conducted two series of reciprocal crosses to assess the inheritance of *bobt*. One set included crosses between individuals carrying *bobt_MV and* individuals without *bobt*, whereas the other set included crosses between individuals carrying *bobt_MV* and *bobt_KR* (Table 1). Presence or absence of each *bobt* allele was assessed using a PCR assay and allele specific primers. In all cases, *bobt* alleles were maternally inherited, reflecting their mitochondrial origin. The conclusive evidence for the mt origin of *bobt* has been obtained recently by 454 sequencing of purified mtDNA in both *MV* and *KR*. The *bobt*-related reads were as abundant as the reads derived from the mt genes, whereas all chloroplast-specific reads were very rare (Muller and Storchova, in preparation).

**Table 1:** Reciprocal crosses between individuals carrying (+) and not carrying (−) *bobt* and individuals carrying *bobt_KR* and *bobt _MV* alleles.

Cross type	Male parent	Female parent	# progeny analyzed	# with *bobt*
**+ and − ** ***bobt***	Kov41 (−)	MV1 (*bobt* +)	12	12
	Kov41 (−)	MV1 (*bobt* +)	12	12
	Kov51 (−)	MV1 (*bobt* +)	12	12
	MV2 (*bobt* +)	Kov45 (−)	10	0
	MV3 (*bobt* +)	Kov45 (−)	10	0
	MV4 (*bobt* +)	Kov45 (−)	10	0
***bobt_MV*** ** and** ***_KR***	MV3 (*bobt_MV*)	KR1 (*bobt_KR*)	0	8
	MV4 (*bobt_MV*)	KR1 (*bobt_KR*)	0	9
	KR2 (*bobt_KR*)	MV1 (*bobt_MV*)	11	0
	KR3 (*bobt_KR*)	MV3 (*bobt_MV*)	10	0

The presence of *bobt* was determined using a PCR assay with allele specific primers.

### Copy numbers of mitochondrial genes

**Figure 2:**
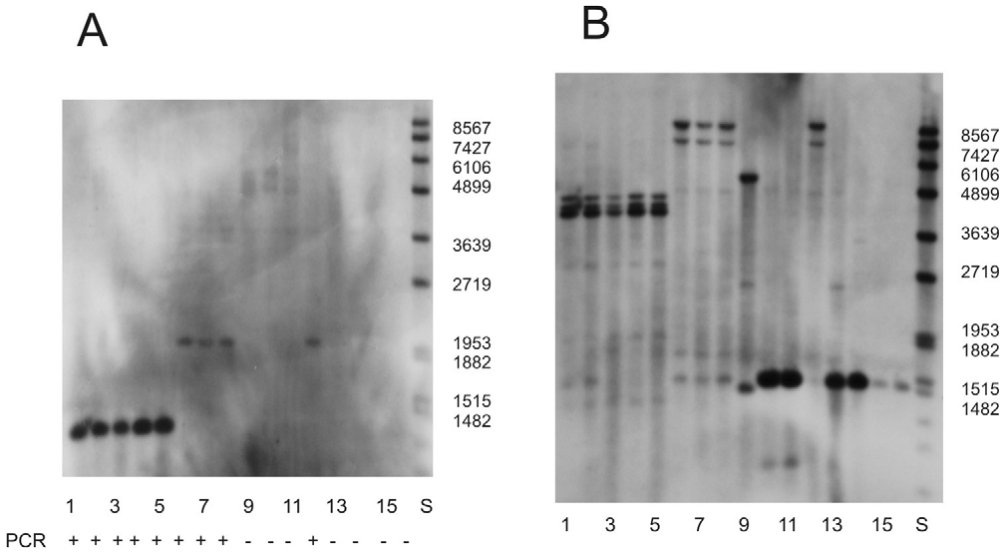
Southern hybridization results with *bobt*-specific and *atp1*-specific probes. Genomic DNA of *Silene* from various sites was digested with EcoRI and hybridized with probes derived from *atp1* and *bobt_MV*. The same membrane was hybridized with both (**A**) an *bobt_MV* probe and (**B**) an *atp1* probe. Lanes 1–5 *S. vulgaris* Mt. View; 6–8 *S. vulgaris* Krasnoyarsk; 9–11 *S. vulgaris* Beagle; 12 *S. vulgaris* Krasnoyarsk; 13 *S. vulgaris* Beagle; 14–16 *S. latifolia* Prague. Individuals from the Beagle population differ in Southern-RFLP pattern of *atp1* region, which is very common in *S. vulgaris* populations. Faint, but visible, bands visible in Beagle and Krasnoyarsk DNA hybridized with a *bobt_MV* probe may correspond to weakly homologous regions in nuclear or mt DNA. The bands corresponding to *bobt_KR* are a bit weaker than *bobt_MV* bands due to nucleotide divergence between the two variants. One band detected by *bobt* probes suggests the existence of only one *bobt* copy in the mt genome, whereas two or three *atp1*-specific bands reflect the existence of two or three *atp1* copies. Marker sizes are shown along the right hand side of each blot. The results of PCR with *bobt* specific primers are shown below (+,−).

Southern hybridization with a probe derived from the coding region unique to *bobt MV* revealed one copy of *bobt* in both the MV and KR mitochondrial (mt) genomes. Stronger hybridization to the MV than the KR genome was caused by higher affinity of the *bobt_MV* probe to MV genome than to KR genome. In *S. vulgaris* DNAs for which we were unable to amplify *bobt* with *bobt*-specific primers, we also found no bands after Southern hybridization (Fig. 2A). When the same membrane was hybridized with an *atp1* specific probe, at least two copies of *atp1* were detected in the KR genome and 3 copies were visible in the MV genome (Fig. 2B); because the conditions were high stringency, none of the *atp1* copies corresponded to the *bobt* gene. As a control, Southern blot studies revealed a single copy of *cytochrome oxidase 1* (*cox1*) in both the KR and MV mt genomes (Fig. S2). The intensities of *atp1-* and *bobt*-specific bands were similar, indicating similar copy numbers for both genes as expected if *bobt* was localized in the mt genome.

**Figure 3:**
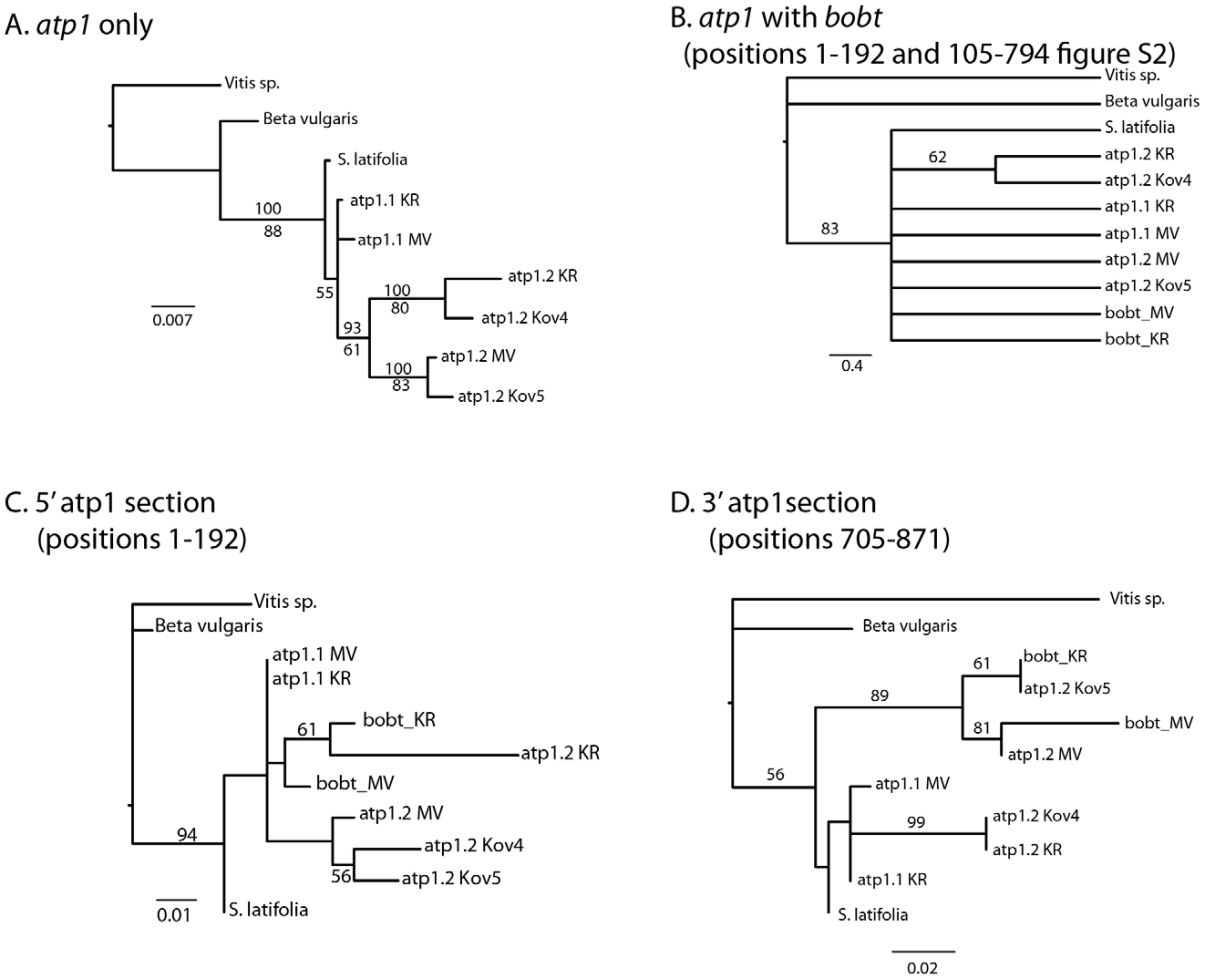
Maximum likelihood phylogenies of *atp1* and homologous regions of *bobt* from *Silene vulgaris* accessions. (A) Phylogeny using the complete sequence of *atp1*. The same topology was recovered when only the regions with homology to *bobt* were used. Numbers above lineages indicate bootstrap support for nodes in the phylogeny with the complete sequence, whereas numbers below the line represent support for node using only regions with homology to *bobt*. (B) Phylogeny including the shared regions of *bobt*, the *atp1* genes found in the same individuals as *bobt* (KR & MV), and two additional atp1 copies from individuals from the Kovary CR population. *Silene latifolia*, *Vitis vinifera*, and *Beta vulgaris atp1* sequences were used as outgroups. (C) Phylogeny of first 192 bases of the shared regions of *bobt* and *atp1* homologous region found at the 5′ end of *bobt*. (D) Phylogeny of the region 3′ to the stop codon of *bobt* and the homologous region in atp1. This phylogeny includes 78 bases than are not found in *bobt_KR*, but are fond in the other accessions.

A single copy was also confirmed for the *cox2* gene in the KR and MV genomes (data not shown). cDNA sequences revealed two different *atp1* variants (*atp1.1* and *atp1.2*) present in both the MV and KR individuals. We tested whether atp1 was duplicated in two individuals that did not carry *bobt*, Kov4 & Kov5; only *atp1.2*, and not *atp1.1*, was present in Kov4 & Kov5. Although this is not an exhaustive sample, it does show polymorphism for the duplication. *Atp1.1* and *atp1.*2 exhibited considerable sequence differences (Fig. S3). A 6 bp insertion/deletion at 3′end of the coding region in both MV and KR backgrounds differentiated *atp1.1* and *atp1.2* and was used to develop version-specific primers for sequencing. *Atp1.1* and *atp1.2* within the MV mitochondria differed in 27 nucleotides, whereas in KR they differed in 35 nucleotides (both excluding the 6 bp indel at 1452–1457; Fig. S3). Phylogenetic analysis indicates that the *S. vulgaris atp1* duplication occurred after divergence from *S. latifolia* (Fig. 3A), but *atp1.1* appears not to be present in all individuals (e.g. Kov4 & Kov5).

### Relationship of *bobt_KR* and *bobt_MV*

The portion of *bobt* homologous to *atp1* coding sequence differed in 10 nucleotides between *bobt_KR* and *bobt_MV* (Fig. 1, both *atp1* segments concatenated; Fig. S1). We anticipated one of two outcomes when we placed the sequences from *bobt* onto a tree with the homologous regions from *atp1*: 1) either both *bobt* alleles would be sister to one another, reflecting a single evolutionary origin and subsequent divergence of *bobt_MV* and *bobt_KR*, or 2) both *bobt* sequences (MV or KR) would be more similar to the homologous sequence in *atp1* from the same individual (MV or KR), a pattern reflecting either recent independent origins of the two *bobt* alleles or persistent and ongoing gene conversion.

Most relationships among sequences were not resolved when homologous regions in *bobt* and *atp1* were analyzed together (Fig. 3B), indicating the introduction of substantial homoplasy into the dataset. To address the origins of this homoplasy, we analyzed the two *atp1* segments present in *bobt* chimeric gene separately. The phylogenetic tree based only on the 5′ *atp1* region of *bobt* (Fig. 1, first *atp1* segment) is depicted in Figure 3C, and the tree based only on the 3′ *atp1* region (Fig. 1, second *atp1* segment) is shown in Figure 3D. Partition homogeneity tests revealed strong incongruence between these regions of *atp1* homology in *bobt* (P = 0.01) and neither phylogeny was consistent with predictions from a single origin of *bobt* without gene conversion (KH tests P<0.001). Interestingly, *bobt_MV* and *atp1.2_MV* shared 78 bp of identical sequence at the end of the region of *atp1* homology 3′ to the *bobt* stop codon (Fig. 1), a likely footprint of recent gene conversion in this small region. The 3′ *atp1* portion of *bobt_KR* is shorter than that of *bobt_MV*, thus any information from this region concerning the relationship of *bobt_KR* to the *atp1* variants has been lost, or was never present.

The *cox2*-derived portions of *bobt_KR* and *bobt_MV* were shorter than portions derived from *atp1* and differed from one another by 4 substitutions (Fig. S1). Whereas three of these were unique to *bobt* (i.e. not present in the functional copies of *cox2*; Fig. S4), the fourth substitution exhibited a difference between *bobt* alleles such that each shared alleles with the corresponding functional *cox2* region in each respective genome (KR or MV, position 231, Fig. S1). These relationships favored either very localized gene conversion or the independent origins of the two allelic versions of *bobt*.

### Expression of *bobt*

**Figure 4:**
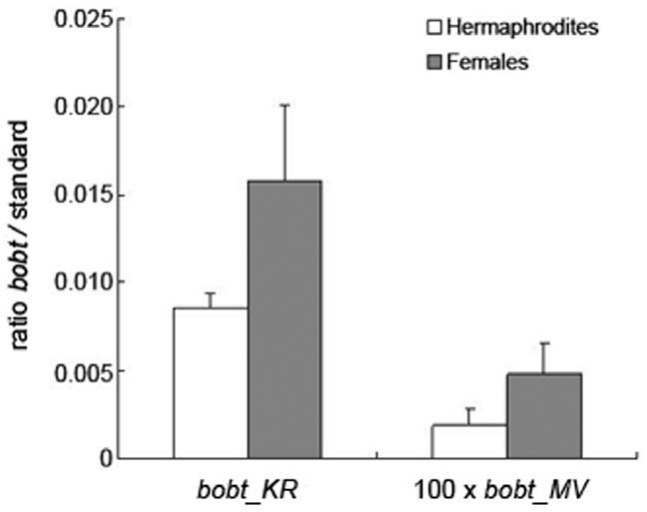
Transcript levels of *bobt_MV* and *bobt_KR* in flower buds estimated by qRT PCR. Expression is presented as a ratio of the accumulation of PCR product for *bobt* and the mt 18S rRNA reference gene. Ratios for *bobt_MV* are multiplied by 100 to allow visualization of difference on the same scale as *bobt_KR*. The standard deviation was calculated on the basis of 3–5 sibling pairs F and H. The differences in *bobt* expression between genders were significant in both MV and KR plants (t-test P<0.05).

Quantitative RT PCR indicated that *bobt* is expressed in both the MV and KR mitochondria, but its expression is over two orders of magnitude higher in KR than in MV (Fig. 4, Fig. S5). PCR experiments indicated that the *bobt* gene is co-transcribed with *cob* in the KR mt genome. Primers targeted to the coding region unique to *bobt_KR* and to *cob* generated a 1220 bp PCR fragment, as expected from the sequence of the *bobt-cob* region (Fig. S6). The *cob* gene is not adjacent to *bobt* in the MV genome, and was not co-transcribed.

**Figure 5:**
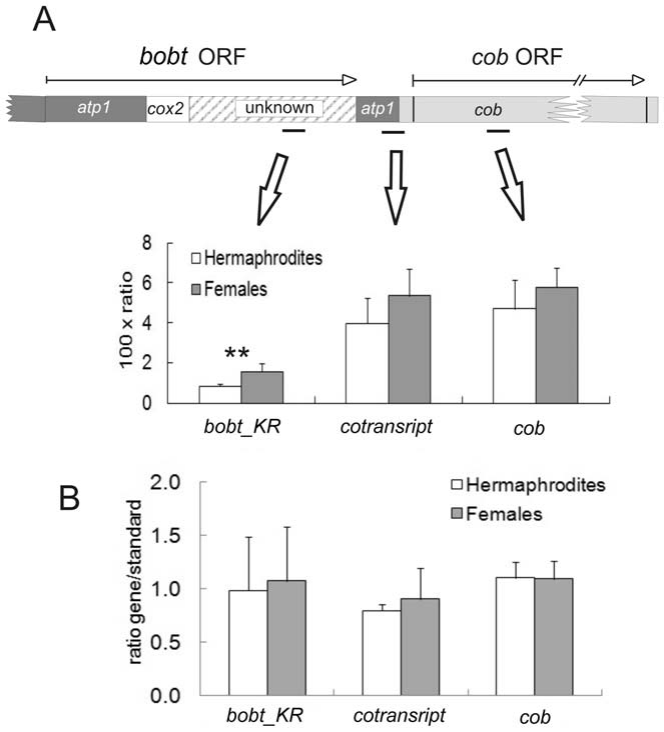
Relative transcript levels in flower buds assessed at different regions of the *bobt_KR*-*cob* co-transcript. (Relative) transcript level at the *atp1-cob* junction and *cob* is more than twice that in the unknown region of *bobt_KR*. No signal was detected when RNA instead of cDNA was added to qPCR reaction mixture, which excluded contamination of RNA samples with genomic DNA. (A) Expression is presented as the ratio of the accumulation of PCR product for the specific region and the mt 18S rRNA reference gene and (B) copy numbers of different regions of *bobt_KR*-*cob* DNA as a ratio of the accumulation of PCR product for the specific region and the mt 18S rRNA reference gene. Whereas target gene and reference copy numbers are approximately equal, *atp1-cob* cotranscript and *cob* transcript levels are more than one order of magnitude lower than mt 18S rRNA. The black lines below the gene indicate the positions of PCR products used for quantitation. The standard deviation was calculated on the basis of 5 sibling pairs F and H. The significant differences (t-test P<0.05) between F and H are marked by **.

Quantification by qRT PCR indicated that *bobt* transcript levels were twice as high in females compared to hermaphrodites in both MV and KR (Fig. 4). Co-transcription of *bobt* and *cob* in the KR genome allowed us to address the stage at which expression was differentially regulated in females and hermaphrodites. qRT PCR studies indicated that *cob* transcript levels were approximately 5 times higher than those of *bobt*. Whereas females had higher *bobt* transcript levels than did hermaphrodite sibs, we detected no significant difference in *cob* transcript levels between female and hermaphrodite sibs (Fig. 5). Moreover, the relative transcript levels measured with qRT PCR primers that targeted the junction between *bobt* and *cob* were similar to the relative transcript levels measured by the primers targeted to the coding region of *cob*. These results indicate that the vast majority of the *cob* transcript in the KR mt genome is derived from the *cob* gene copy co-transcribed with *bobt* and not from other copies of *cob* in the KR genome, if they are there. To exclude the possibility that differences in transcript levels reflected differences in gene copy number and not altered expression, we performed qRT-PCR with genomic DNA as a template under exactly the same conditions as measurements with cDNA. Copy numbers of all the analyzed regions appeared to be equal (Fig. 5B). Taken together, these data indicate that *bobt* expression is lower in hermaphrodites than in females in KR, whereas *cob* transcript levels are approximately equal in both genders. We speculate that a post-transcriptional process is responsible for this phenomenon.

### Post-transcriptional editing of *bobt*

Comparing direct sequences from 3 cDNA PCR products to the genomic sequence in *bobt* (also directly sequenced) revealed that one site was edited in the *atp1* region (position 784 Fig. S1) and one site was edited in the *cox2* region of *bobt* (position 249 Fig. S1) for both the *bobt_KR* and *bobt_MV* alleles. These sites also were edited in the functional copies of *atp1.2* and *cox2* (Figs. S1 and S4) indicating that the signal for editing was transferred with the sequence when intragenomic recombination created *bobt*.

## Discussion

This study provides unique insight into evolution of chimeric genes in plant mitochondria. Our observation of high divergence in nucleotide sequence of the two alleles of a complex chimeric gene, *bobt_MV* and *bobt_KR*, is surprising. We know of one other study, in *Raphanus raphanistrum* [21], that observed two alleles in a chimeric mt gene, but these differences were much less striking than what we found in *S. vulgaris*. One characteristic of *S. vulgaris* that may have contributed to high divergence between these alleles was the high mitochondrial DNA mutation rate in this species, which also influences the high diversity found in the *atp1* gene [29], [44], [45]. Overall, 43 single nucleotide differences were observed between *bobt_MV* and *bobt_KR*, counting from the start codon through the 3′ regions homologous to *atp1*; 10 of these differences were within the segments homologous to *atp1* (Figs. 1 and S1).

The divergence between the two *bobt* alleles and the *atp1* variants in *S. vulgaris* allowed us to test whether *bobt* had a single evolutionary origin or two independent origins. The phylogenetic analysis of *atp1* genic segments of *bobt* and the corresponding regions of the *atp1* genes did not provide an unequivocal solution. Although it is possible that *bobt* evolved twice independently, given the complex structure of this chimeric gene and the presence of a large region with unknown homology, it is more parsimonious to assume a single origin of the gene. Assuming a single origin, the two *bobt* alleles have taken remarkably different evolutionary routes that include nucleotide divergence likely influenced by gene conversion and structural re-arrangement of flanking regions that have influenced expression patterns. In particular, our study contributes to the recent realization of the commonness of gene conversion in angiosperm mitochondria [46] that is motivating reassessment of the factors contributing to this genomes' evolution [47].

Gene conversion also likely contributed to divergence between *bobt_MV* and *bobt_KR*. Its affect can be most easily seen in the nearly identical sequences from sites 780–848 for *atp1.2_MV* and *bobt_MV* in Figure S1. Several instances of gene conversion in plant mitochondrial genes have been described recently [46], [48], [49] and patterns in *bobt* indicate that gene conversion must be a persistent characteristic of its evolution in *Silene vulgaris*. Moreover, independent gene conversion events appear to have occurred in each *bobt* lineage, generating patterns consistent with the phylogeny in Figure 3C.

The overall structure of *bobt* reveals clues regarding its origin. Chimeric ORFs are created from the fusion of DNA fragments via recombination across shared short repeats (<30 bp). Recombination at these short repeat sites in plant mitochondria is considered very rare and generally they are not considered to facilitate homologous recombination (Marechal and Brisson 2010), despite some recent findings that even very short repeats (6 bp) can mediate recombination [50]. We found an 8 bp region of complete identity at the junction between the *atp1* and *cox2* segments of *bobt*, which is a likely site for repeated recombination between the *atp1* and *cox2* genes. Because the origin of the internal part of the *bobt* genes is unknown, we are unable to identify possible regions of similarity and potential recombination between the internal unknown region and the segments of *cox2* and *atp1*. The presence of hotspots of recombination may increase the probability of independent origins of the two *bobt* alleles (*bobt_KR* and *bobt_MV*). We note that the same *cox2* segment has been independently incorporated into two different chimeric regions in petunia and wheat, although the wheat region is likely a pseudogene [51], [52], [53].

Although not detected in previous studies [44], [54], here we documented multiple copies of *atp1* within the MV and KR genomes. The dynamic nature of angiosperm mt genomic rearrangements has been shown to result in dramatically different copy numbers and relative positions of genes within genomes [55], [56], [57]. It is likely that *Silene* also harbors high variation in gene copy number among lineages and this is a likely explanation for the absence of previous detection. Nonetheless, caution is justified when assuming homology among mt coding regions in *Silene* and other plant species.

*Bobt* has many of the hallmarks of CMS genes. One commonality among most chimeric CMS genes is the presence of portions of *ATP synthase* subunits [1]; portions of these subunits are found in both *bobt* alleles. CMS genes also tend to be transcribed; they are not loss-of-function genes. Both *bobt* genes are transcribed, albeit at a comparably low level in the case of *bobt_MV*. Finally, nuclear restorers of fertility (Rf) often post-transcriptionally interfere with the products of CMS genes, so that hermaphrodites accumulate less transcript than females [58], [59], [60]. Both *bobt* alleles exhibited lower transcript levels in hermaphrodite than in female plants. The much higher transcript level for *bobt_KR* than for *bobt_MV* in both female and hermaphrodite plants, however, may indicate significant differences in the functionality of these alleles including the possibility that *KR_bobt* is a CMS-related gene but *MV_bobt* is not. The different genomic context between *bobt_KR* and *bobt_MV* is likely related to the difference in transcript levels. *bobt_KR* is co-transcribed with *cob*, which lacks its own promoter, although a start of transcription from a sequence motif located upstream of the *cob* coding region, including the unknown region of *bobt*, cannot be completely excluded. In contrast, *bobt_MV* is not co-transcribed with *cob*. Although additional *cob* copies may be present in Krasnoyarsk plants, the high transcript level detected at the junction between *bobt_KR* and *cob* suggests that the co-transcript is a major source of *cob* mRNA. We may speculate that co-transcription plays a role in the inability to independently regulate transcription of *cob* and *bobt_KR*, however, our studies also show that *bobt* might be post-transcriptionally regulated independently from *cob*. We are curious to know whether the co-transcription may limit the options for new mutations to arise that down-regulate transcription of *bobt_KR* and other CMS-associated genes. Co-transcription of CMS-associated genes with essential protein coding genes is not unique to the present study. For example, o*rf456* is co-transcribed with *cox2* in chili pepper [60], *orf256* is co-transcribed with *cox1* in wheat [61], and a CMS-related factor is co-transcribed with *nad6* in *Mimulus* [62]. Molecular options for suppression of expression may be more limited when CMS genes are co-transcribed with housekeeping genes.

Our study indicates that highly divergent copies of complex chimeric genes with similar structures as CMS genes are present within the same species, albeit in individuals with wide geographic separation. The ecology and evolution of gynodioecious mating has been studied in *S. vulgaris* for many years and multiple studies have remarked on the high level of mitochondrial gene polymorphism [22], [44], [45], especially when compared to hermaphroditic congeners [22]. Discussion of the source of mitochondrial divergence has been primarily whether it results from high mutation rates within gynodioecious species or balancing selection maintaining divergent copies over long time periods. Our study suggests that gene conversion may also be a process contributing to divergence. Combined with the increasingly convincing evidence for low frequency paternal inheritance of mitochondrial variation [63], gene conversion between two copies of a duplicated gene provides increasingly complex possibilities for the origins of plant mitochondrial genes. We should not let this complexity divert us from conducting carefully studies to determine the tempo and mode of plant mitochondrial genome evolution.

In summary, we have discovered two divergent allelic versions of a complex mitochondrial chimeric ORFs in different *S. vulgaris* individuals. Comparisons between homologous regions between the chimeric genes and *atp1* are consistent with a history of independent gene conversion events in each lineage. Gene expression also has diverged both in quantity and the co-transcription of an adjacent *cob* gene in the *bobt_KR* version. These observations indicate that chimeric genes may persist for sufficiently long periods within species for significant evolution in nucleotide sequence, expression and genomic environment.

## Supporting Information

Figure S1 **The alignment of** ***bobt*** **genes and the corresponding region of** ***atp1*** **variants from KR and MV genomes.** The alignment begins with the putative regulatory region upstream start codon, followed by a long insertion unique to *atp1.2_KR*. The putative regulatory region has not yet been sequenced for *atp1.1_KR*, which starts at position −46 in this figure. Regions of complete homology among all genes are shown in yellow and regions with substitutions or lack of complete homology are shown in blue. The region of homology between *cox2* and *bobt* is shown in magenta. Note the 8 bp regions of homology between atp1 and cox2, which is a likely site for the recombination events that created *bobt*. This region of homology is followed by another motif highly similar between *atp1* and *cox2*. Sites of post-transcriptional editing are marked with an E.
(TIF)

Figure S2 **Southern hybridization with the** ***cox1*** **probe.** Genomic DNA of *Silene* from various locations was digested with EcoRI and hybridized with probes derived from *cox1.* 1–5 *S. vulgaris* Mt. View; 6–8 *S. vulgaris* Krasnojarsk Czech Republic; 9–11 *S. vulgaris* BeagleVirginia, USA; 12 *S. vulgaris* Krasnojarsk; 13 *S. vulgaris* Beagle Virginia, USA; 14–16 *S. latifolia* Prague, Czech Republic. In addition to the single major *cox1* copy, faint bands corresponding to the band in the individual from Beagle (line 9) are visible in the plants from Mt.View and Krasnojarsk. They may represent *cox1* variants present in low copy number.
(TIFF)

Figure S3 **Alignment of sequences of** ***atp1*** **variants in** ***S. vulgaris*** **. Outgroup sequences were** ***atp1*** **from** ***S. latifolia*** **(GenBank acc. No. HM099771),** ***Beta vulgaris*** **(AB007034).**
(TIF)

Figure S4 **The alignment of** ***cox2*** **genes in MV and KR genomes based on partial coding sequences.** An editing site is marked by E.
(TIFF)

Figure S5 **Agarose gel showing PCR and RT PCR amplification using the primers 21 and 22 (Table S1), that are specific for** ***bobt_MV*** **.** Lanes 1, 2 – PCR using DNA extracted from the leaves of two *S. vulgaris* individuals from Mt View; lanes 3, 4 – RT-PCR conducted on total RNA extracted from the flower buds of the same *S. vulgaris* Mt View plants which were used to prepare DNA amplified in the first two lanes; lanes 5, 6 – negative controls – PCR with total RNA from the same two *S. vulgaris* Mt View individuals as used before. Marker sizes are shown at right hand side of the gel.
(TIFF)

Figure S6 **PCR confirmation of** ***bobt_KR-cob*** **co-transcription. cDNAs of** ***S. vulgaris*** **Mt.View (1 and 5) and Krasnojarsk (2–4) were PCR amplified with** ***cob*** **and** ***bobt*** **specific primers.** A 1220 bp fragment is shown in the gel image and was produced only in the samples from Krasnojarsk.
(TIFF)

Figure S7 **Locations of primers for sequencing** ***bobt_KR*** **and** ***bobt_MV.***
(TIFF)

Table S1 **Primers used in this study.**
(PDF)
