# Supplementary figures and images for: Mosaic Origins of a Complex Chimeric Mitochondrial Gene in Silene vulgaris
Source: PLoS One. 2012 Feb 27;7(2):e30401. doi: 10.1371/journal.pone.0030401 (PMC3288002; doi:10.1371/journal.pone.0030401)

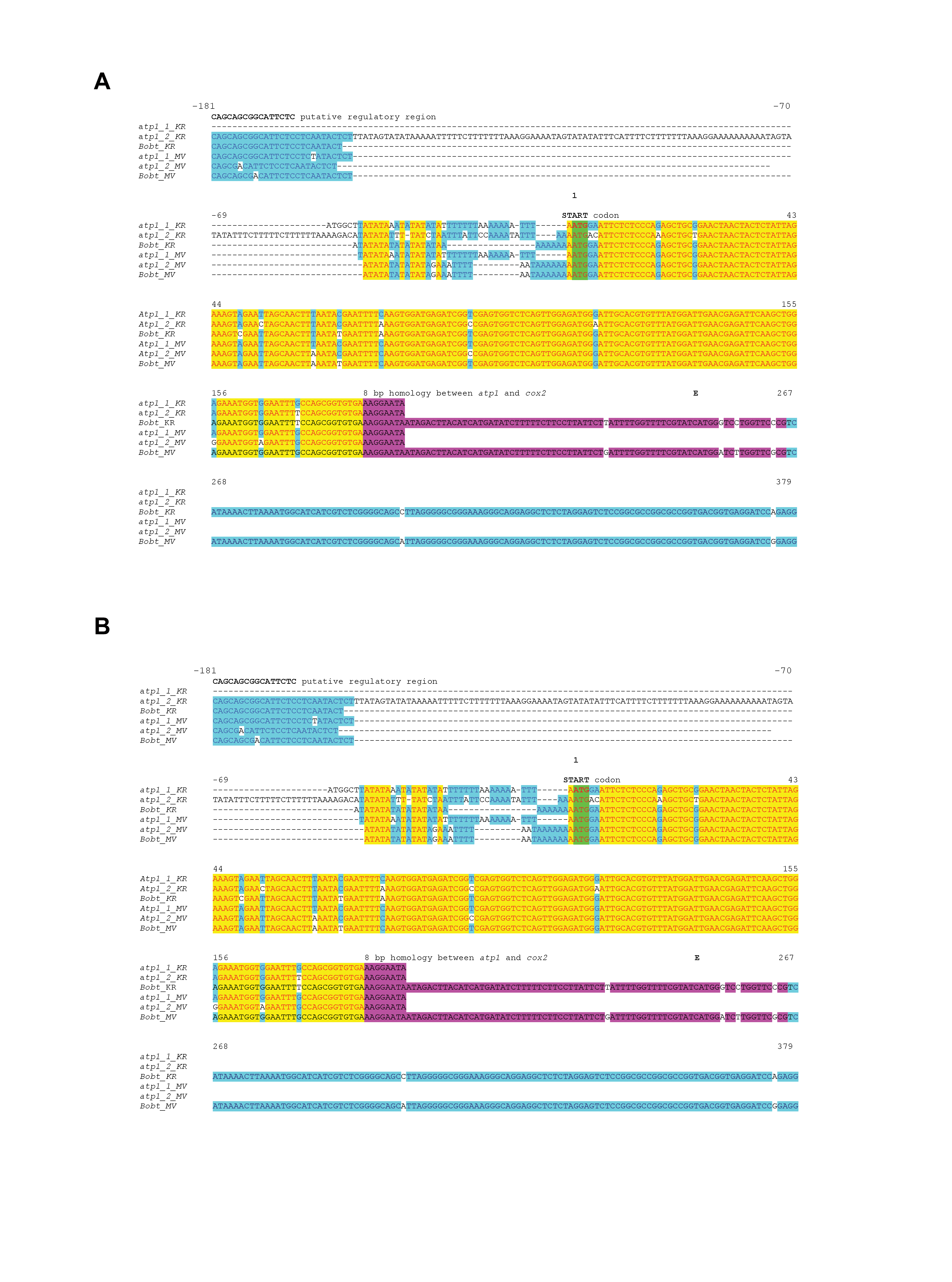

Supplement: Figure S1 — The alignment of bobt genes and the corresponding region of atp1 variants from KR and MV genomes. The alignment begins with the putative regulatory region upstream start codon, followed by a long insertion unique to atp1.2_KR. The putative regulatory region has not yet been sequenced for atp1.1_KR, which starts at position −46 in this figure. Regions of complete homology among all genes are shown in yellow and regions with substitutions or lack of complete homology are shown in blue. The region of homology between cox2 and bobt is shown in magenta. Note the 8 bp regions of homology between atp1 and cox2, which is a likely site for the recombination events that created bobt. This region of homology is followed by another motif highly similar between atp1 and cox2. Sites of post-transcriptional editing are marked with an E. (TIF) [file pone.0030401.s001.tif]

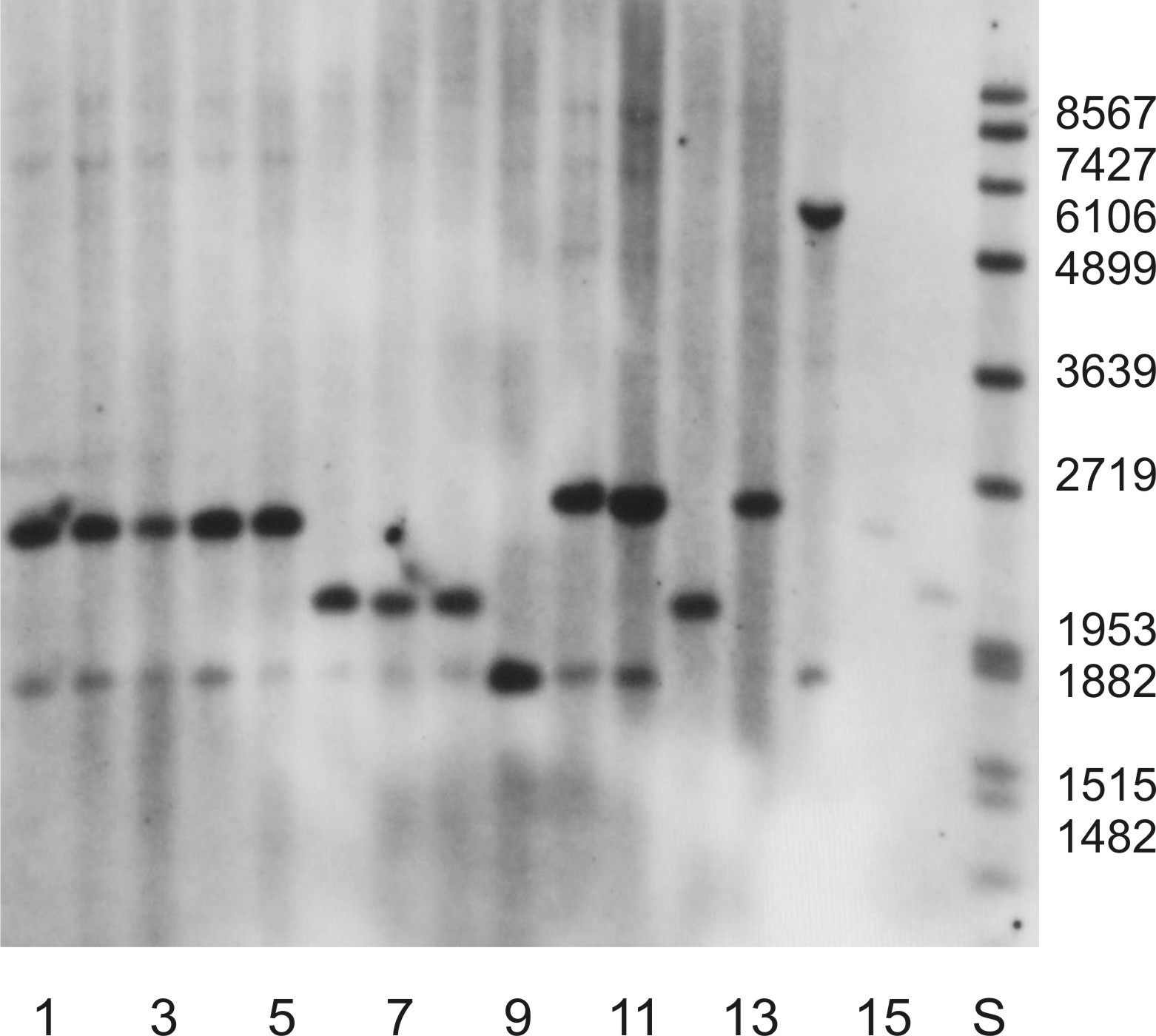

Supplement: Figure S2 — Southern hybridization with the cox1 probe. Genomic DNA of Silene from various locations was digested with EcoRI and hybridized with probes derived from cox1. 1–5 S. vulgaris Mt. View; 6–8 S. vulgaris Krasnojarsk Czech Republic; 9–11 S. vulgaris BeagleVirginia, USA; 12 S. vulgaris Krasnojarsk; 13 S. vulgaris Beagle Virginia, USA; 14–16 S. latifolia Prague, Czech Republic. In addition to the single major cox1 copy, faint bands corresponding to the band in the individual from Beagle (line 9) are visible in the plants from Mt.View and Krasnojarsk. They may represent cox1 variants present in low copy number. (TIFF) [file pone.0030401.s002.tiff]

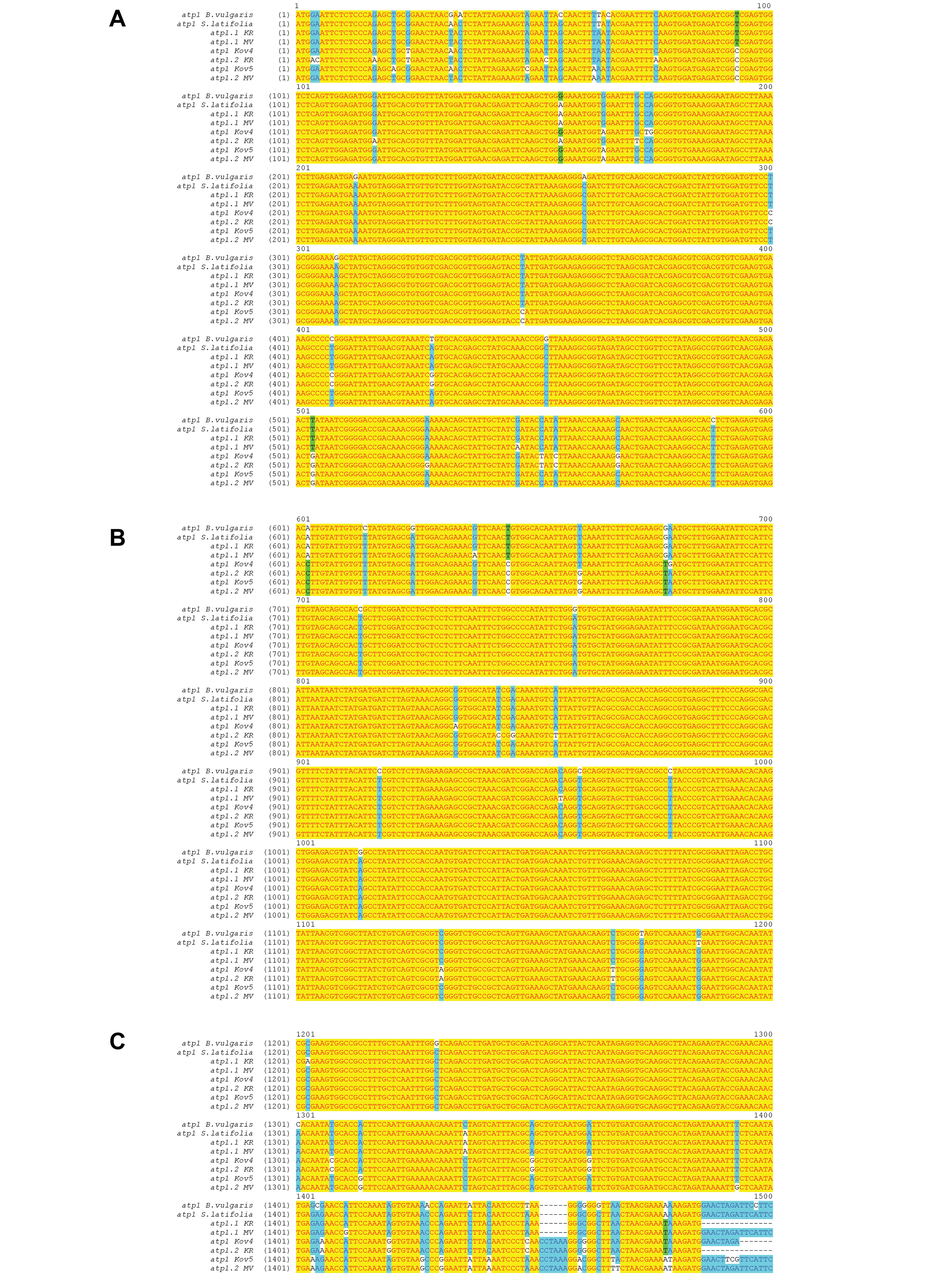

Supplement: Figure S3 — Alignment of sequences of atp1 variants in S. vulgaris . Outgroup sequences were atp1 from S. latifolia (GenBank acc. No. HM099771), Beta vulgaris (AB007034). (TIF) [file pone.0030401.s003.tif]

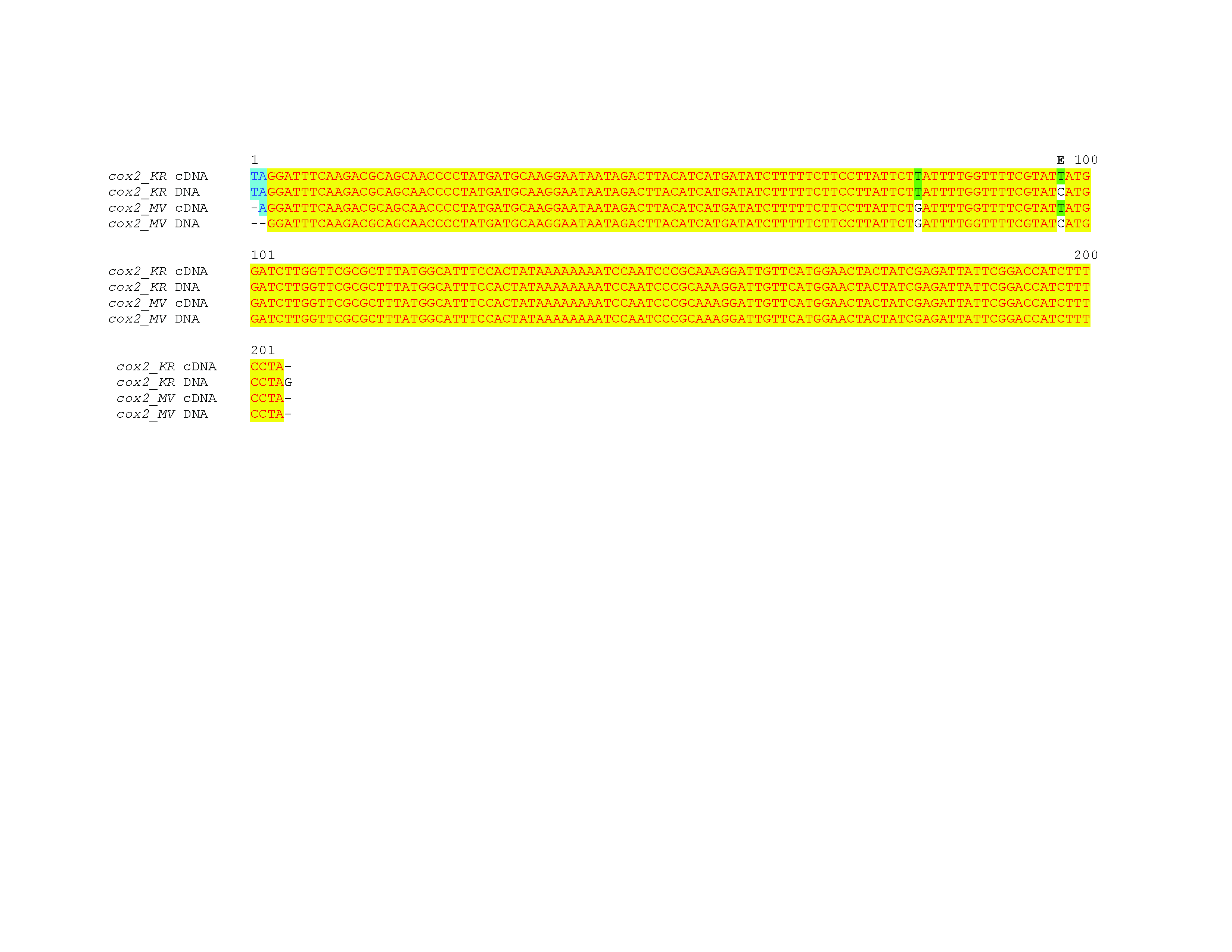

Supplement: Figure S4 — The alignment of cox2 genes in MV and KR genomes based on partial coding sequences. An editing site is marked by E. (TIFF) [file pone.0030401.s004.tiff]

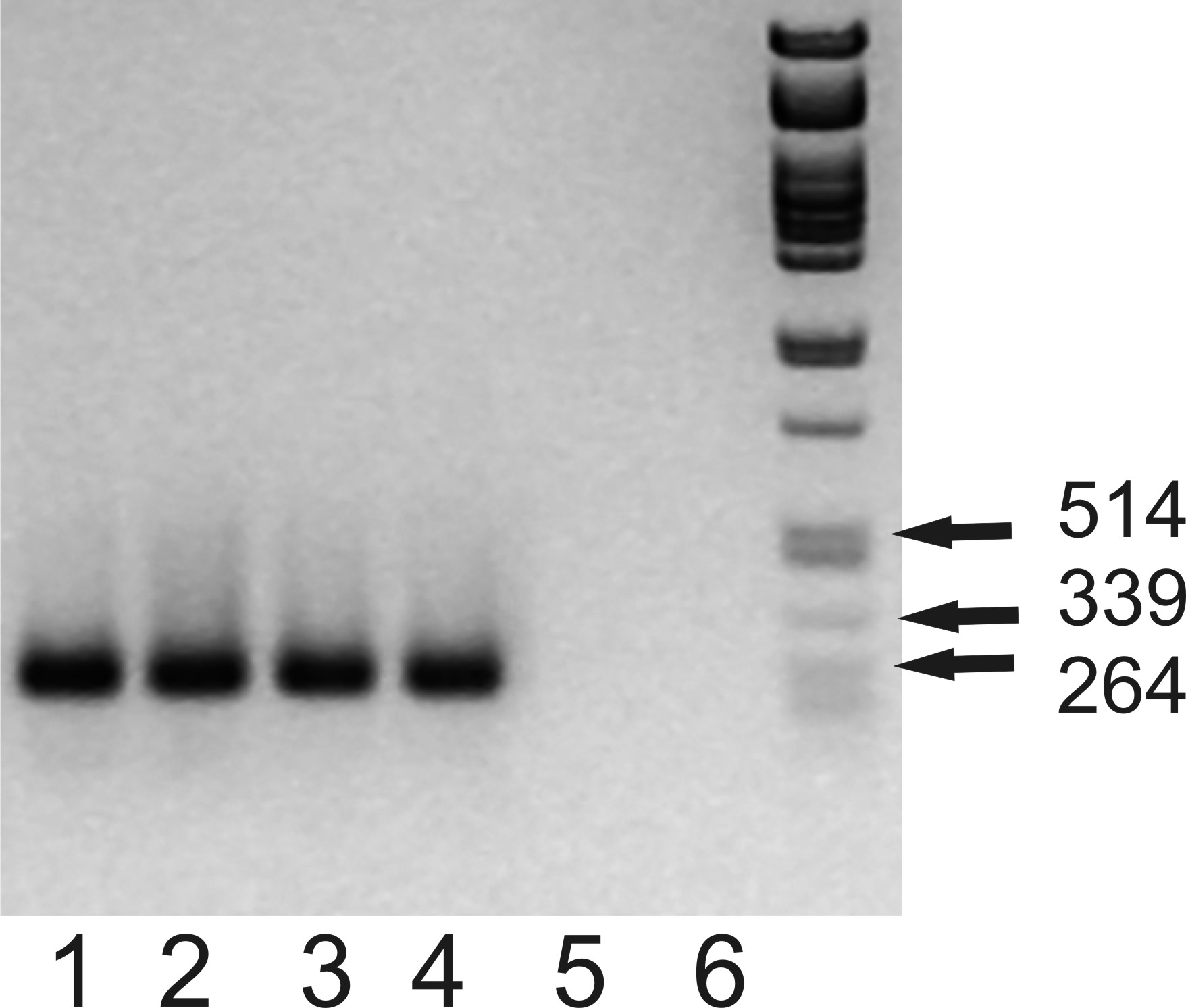

Supplement: Figure S5 — Agarose gel showing PCR and RT PCR amplification using the primers 21 and 22 (Table S1), that are specific for bobt_MV . Lanes 1, 2 – PCR using DNA extracted from the leaves of two S. vulgaris individuals from Mt View; lanes 3, 4 – RT-PCR conducted on total RNA extracted from the flower buds of the same S. vulgaris Mt View plants which were used to prepare DNA amplified in the first two lanes; lanes 5, 6 – negative controls – PCR with total RNA from the same two S. vulgaris Mt View individuals as used before. Marker sizes are shown at right hand side of the gel. (TIFF) [file pone.0030401.s005.tiff]

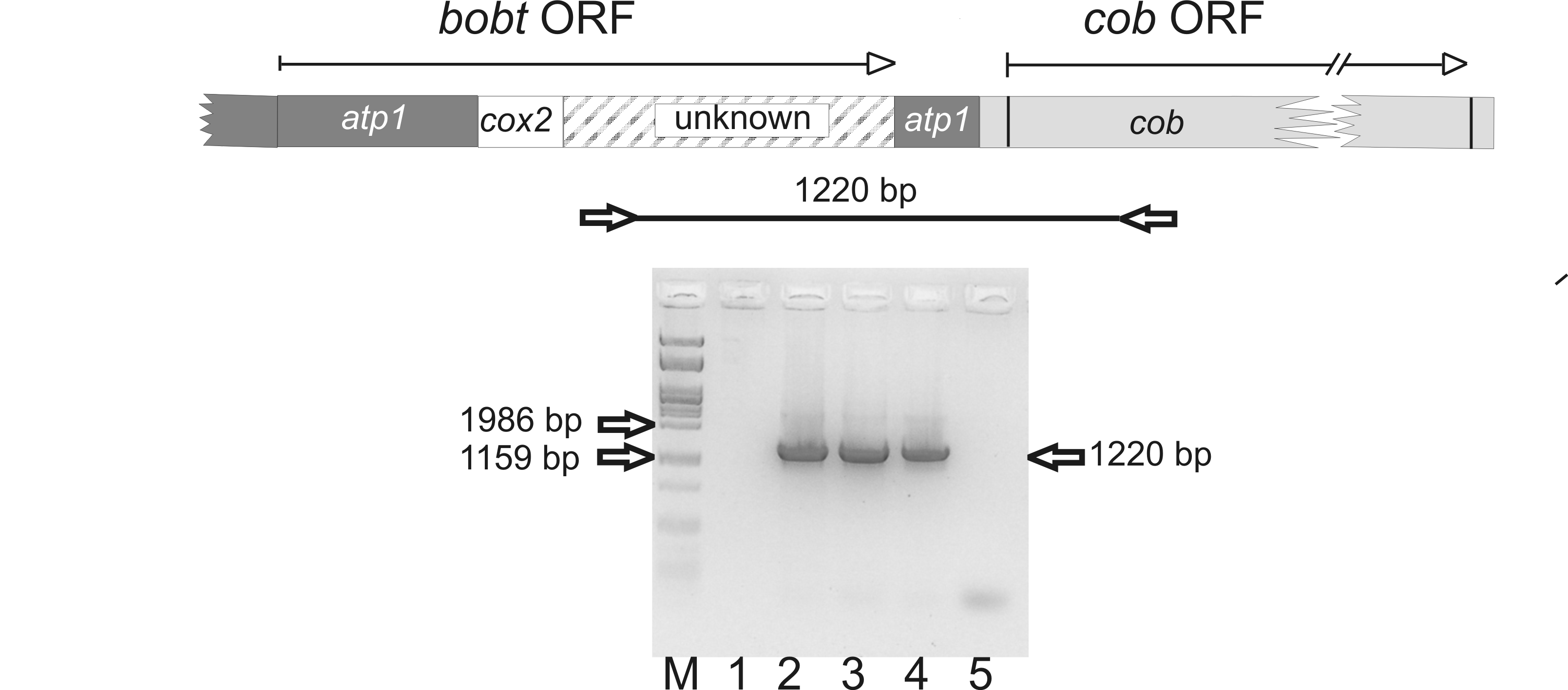

Supplement: Figure S6 — PCR confirmation of bobt_KR-cob co-transcription. cDNAs of S. vulgaris Mt.View (1 and 5) and Krasnojarsk (2–4) were PCR amplified with cob and bobt specific primers. A 1220 bp fragment is shown in the gel image and was produced only in the samples from Krasnojarsk. (TIFF) [file pone.0030401.s006.tiff]

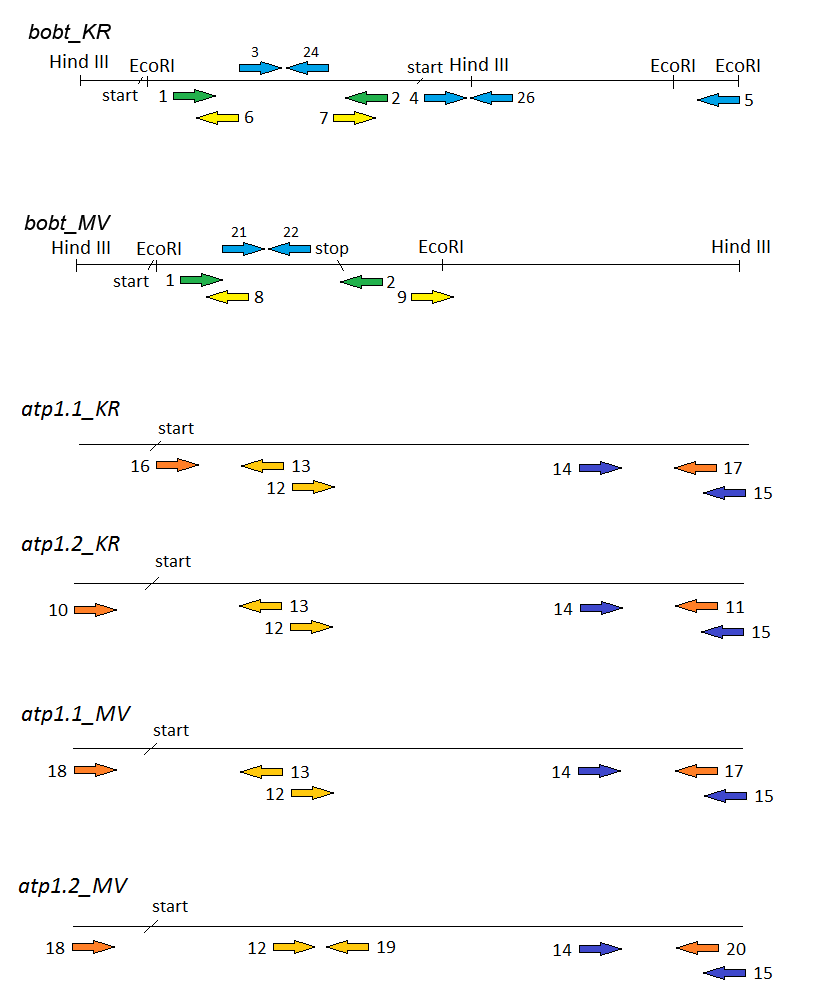

Supplement: Figure S7 — Locations of primers for sequencing bobt_KR and bobt_MV. (TIFF) [file pone.0030401.s007.tiff]
