# Supplementary material for: Mosaic Origins of a Complex Chimeric Mitochondrial Gene in Silene vulgaris
Source: PLoS One. 2012 Feb 27;7(2):e30401. doi: 10.1371/journal.pone.0030401 (PMC3288002; doi:10.1371/journal.pone.0030401)
Supplement: Table S1 — Primers used in this study. (PDF) [file pone.0030401.s008.pdf]

Table S1. Primers used in this study.

Primer Class	Primer Name	Gene/location	Sequence
Sequencing	atpA up	Primer 1, Fig S7	TACACGAATTTTCAAGTGGATGA
	atpA lo	Primer 2, Fig S7	TCTAGTGGCATTCGATCACAGA
	BobKr 188For	Primer 3, Fig S7	GTCCTGGTTCCCGTCATAAA
	cob 253F	Primer 4, Fig S7	TTGGGGTCAGATGAGCTTTT
	cob R2	Primer 5, Fig S7	TCAATTCTTGGTGTAAGAAT
	InvBobAtp Rev2	Primer 6, Fig S7	CATGATACGAAAACCAAAATCAGA
	InvBobAtp For1	Primer 7, Fig S7	TTACGCAGCTGTCAATGGAT
	InvBobAtp Rev3	Primer 8, Fig S7	GCAATCCCATCTCCAACTGA
	InvBobAtp For2	Primer 9, Fig S7	TTCGCAGGACTATCTTCTTTATC
	KrBob For2	Primer 10, Fig S7	TCTTCTCGTACTCTTGCAGCA
	Atp1 3'end cDNA rev2	Primer 11, Fig S7	CCCCCTTTAGGTTGAGGGATT
	Atp297F	Primer 12, Fig S7	TCGACGTGTCGAAGTGAAAG
	Atp271R	Primer 13, Fig S7	GACCACACGCCCTAGCATA
	Atp1end For2	Primer 14, Fig S7	TGAAACAAGTCTGCGGGAGT
	Atp1end Rev2	Primer 15, Fig S7	AAGAATGAATCTAGTTCCATC
	Atp1 MtV Start For	Primer 16, Fig S7	ATGGAATTCTCTCCCAGAGC
	atp1 3'end DNA rev2	Primer 17, Fig S7	AGCCGCCCTTTAGGGATTGTA
	MtVBob For1	Primer 18, Fig S7	CTTTCCTTATGTCCAATCAGCA
	atp 587R	Primer 19, Fig S7	ACGGTTGAACGTTTCTGTCC
	Atp1 3'end cDNA rev1	Primer 20, Fig S7	CGTCCTTTAGGTTTAGGGATT
qRT PCR	putORF atpA56F2	*bobt_MV*	GGCAGGAGGCTCTCTAGGAG
	putORF atpA279R2	*bobt_MV*	TGGATTCAATGTTGTCCGTGAC
	BobKr_188For	*bobt_KR*	GTCCTGGTTCCCGTCATAAA
	BobKr_393Rev	*bobt_KR*	GACCGCATTTGGCATCTG
	cob253F	*cob*	TTGGGGTCAGATGAGCTTTT
	cob443R	*cob*	GGCCAGATGAAGAAGACTGG
	InvBobAtp For1	*co-transcript*	TTACGCAGCTGTCAATGGAT
	BobKr cotr Rev2	*co-transcript*	AATCGTTGGTTCCTTAGACTCAT
	SVB28 91F	*18S rRNA*	GCTGTCAGACTGAGTCTTTG
	SVB28 243R	*18S rRNA*	GTCTTCAAATAGGGCGAGAA
probes	atpA lo	*atp1*	TCTAGTGGCATTCGATCACAGA
	atpA up	*atp1*	TACACGAATTTTCAAGTGGATGA
	BobKr_188For	*Bobt_KR*	GTCCTGGTTCCCGTCATAAA
	BobKr_393Rev	*Bobt_KR*	GACCGCATTTGGCATCTG
	Cob IP 59	*cob*	GATTATCCAACCCCGAGC
	Cob IP 64	*cob*	GAATGGGCGTTATGGC
